# Classification system of ultrasound patterns of non-nodular thyroid diseases

**DOI:** 10.1007/s12020-025-04460-z

**Published:** 2025-10-15

**Authors:** Pierpaolo Trimboli, Jörg Bojunga

**Affiliations:** 1 Thyroid Unit, Clinic for Endocrinology and Diabetology, Ente Ospedaliero Cantonale (EOC), Bellinzona, 6500 Switzerland; 2 Faculty of Biomedical Sciences, Università Della Svizzera Italiana, Lugano, 6900 Switzerland; 3 Department of Medicine I, Goethe University Hospital, Theodor-Stern-Kai 7, Frankfurt am Main, 60590 Germany

**Keywords:** Thyroid, Ultrasound, Classification, TIRADS

## Abstract

**Purpose:**

Thyroid disorders are largely present in the general population. In addition to the medical history and laboratory tests, ultrasound (US) is a sensitive method for assessing thyroid diseases. Since thyroid nodules (TNs) are significantly more prevalent than non-nodular diseases – i.e., autoimmune, inflammatory, etc. – and entirely normal thyroid pattern, the largest part of the specific literature has been focused on TN. Indeed, international societies have developed systems to stratify the cancer risk of TN (i.e., TIRADS) while no classification exists to assess non-nodular disorders.

**Methods:**

A three-score US-based classification of thyroid parenchyma is proposed: (1) pattern “N” – normal parenchyma, (2) pattern “A” – autoimmune - to assess autoimmune disease; (3) pattern “I” – inflammation - to assess thyroid inflammation/infection diseases.

**Results and conclusions:**

Such a system could (1) cover the current lack of standardized classification of parenchyma, (2) improve the accuracy of US operators, and (3) facilitate the exchange among thyroidologists (endocrinologists as first) and stakeholders.

## Background – epidemiological notes

Large part of general population suffers from thyroid disorders. Thyroid disease is common with a estimated prevalence of thyroid nodule (TN) ranging from 10% up to 70% [1], and incidence rate for thyroid cancer (TC) of 2.16–6.6/100.000 [2], and autoimmune thyroid disorders (ATD) from 25–100/100.000 of Graves’ disease (GD) to 100–250/100.00 of Hashimoto thyroiditis (HT) [3, 4], respectively. In addition to the medical history and laboratory tests, ultrasound (US) is the most sensitive method for diagnosing thyroid diseases. A final diagnosis can usually be made based on these findings. The quality and standardization of US findings is therefore particularly important. Usually, the use of B-mode US is sufficient; however, echo-color doppler provides information on parenchymal vascularization and may be helpful for differentiating diffuse thyroid disease from normal pattern [5].

When assessing the incidence of thyroid disease, methodological aspects must be considered alongside the epidemiological ones. The largest part of epidemiological studies adopted neck palpation or laboratory tests to furnish data about the prevalence of thyroid diseases. Also, US examination has been generally used only in specific settings to analyze the prevalence of nodular goiter. Distinguishing nodular from diffuse thyroid diseases is of relevance here, as both introduction of screening programs [6] and increase in thyroid US examinations [7] has led to a massive overdiagnosis of nodules and carcinomas, mostly very small papillary carcinoma (PTC). Nevertheless, the prevalence of PTC in autoptic studies remained stable over six decades [8]. In the period from 1998 to 2012, the rapid increase in thyroid cancer incidence were observed only for PTC, a type more likely to be found in a subclinical form and therefore detected by intense screening [9]. The current thyroid cancer epidemiological landscape is strongly suggestive of a large effect of overdiagnosis in many countries and settings worldwide, confirming the relevance of PTC overdiagnosis as a global public health problem [10]. Age, iodine deficiency status, and other factors can also influence these estimates. The age-standardized prevalence of thyroid disease among American adults increased from 1999 to 2003, remained stable between 2003 and 2014, and then saw an increase from 2014 to 2018, with the highest rate observed among elders, women, and non-Hispanic whites [11]. In a study from Italy [12], the incidence of HT decreased from 15.86 in 2013 to 12.35 in 2022. The decline was evident only in females and was more pronounced in females aged 15–54 years than in those aged ≥55 years. In 2020, an out-of-trend decrease in HT incidence was documented, corresponding with the SARS-CoV-2 pandemic, with a realignment to the trend in the subsequent years. A decline in ATD was documented in the Italian Veneto region in the last decade, paralleling improvement in the iodine status. In a German long-term observation, a decreased prevalence of iodine-deficient disorders and a stable prevalence of markers of autoimmune thyroid disorders were observed and argue for an improved iodine supply of the adult population in Northeast Germany [13]. In contrast, prevalence of diagnosed thyroid disorders and intake of thyroid medication increased, although this might be related to inappropriate therapeutic decisions. The program of iodine fortification (IF) in Denmark followed two Danish sub-populations in the years before IF and up till 20 years after [14]. The level of thyroglobulin and thyroid volume decreased following IF, and the incidence of TN lowered. The incidence of hyperthyroidism increased transiently following IF but subsequently decreased below the pre-fortification level. Conversely, TSH levels and prevalence of ATD increased along with an increase in the incidence of hypothyroidism. These trends were mirrored in the trends in treatments for thyroid disease.

## Current use of thyroid US and lacking data

Since TNs are significantly more prevalent in clinical practice than non-nodular diseases and the normal thyroid pattern, considering that both clinicians and researchers are much more interested in TNs with potential to be TC, the largest part of thyroid US literature has been focused on TNs. Alongside the laboratory thyroid function tests, US examination is largely used in the initial clinical assessment of patients with suspected TN [15]. Indeed, US is recognized as pivotal to stratifying the risk of malignancy of TNs and then indicate biopsy when necessary. Because of the very good performance of US in detecting TN and selecting them for biopsy or follow-up, and considering the important prevalence of TN (and as a consequence of TC) in the general population, international societies have developed specific US-based classification systems with the aim to (1) standardize the lexicon for reporting TN discovered at US; (2) facilitate their risk assessment; and (3) improve the selection of cases requiring biopsy by reducing the unnecessary ones. As the reliability of these systems, usually defined with the acronym TIRADS (Thyroid Imaging Reporting And Data System), has been largely proven in the literature, they were rapidly adopted in clinical practice by US operators, firstly endocrinologists [16]. Nevertheless, while both HT and GD occur with a non-negligible rate especially in adults and elderly, and bearing in mind that patients with TNs may have also autoimmune-based dysfunction, it is remarkable that no classification system exists to assess non-nodular diseases. In addition, other generally non-nodular diseases such as subacute-De Quervain thyroiditis (SAT) are ignored by TIRADSs and can be difficult to be interpreted by thyroid-inexperienced US operators representing a cause of misleading reports and unnecessary biopsy. Lastly, apart from rare exceptions, we currently have no standardized modality to assess thyroid gland as normal in cases where its echostructure appears as regular in the absence of nodular lesions/echogenicity alterations. In fact, some TIRADSs includes a category of normal thyroid (i.e., class 1 of EU-TIRADS [17] and the TIRADS described by Horvath et al. [18]) while there is no class to assess non-nodular thyroid disease. Thus, it seems that thyroid US classification systems were conceived to classify thyroids as nodular – with estimation of cancer risk – or normal without other details.

## Purpose of the document

Many clinical thyroidologists perform thyroid US during their visit. However, thyroid US can also be performed in US point of services by radiologists who could be unaware of thyroid status, clinical context, and patient’s history. Furthermore, these examinations may be conducted by personnel with insufficient experience in the thyroid field. In addition, reports of neck US examinations conducted according to non-thyroidal indication often include marginally the description of the thyroid. Interpreting these results, mostly without images/clips, can lead to diagnostic errors and inappropriate case management. Having a classification system for non-nodular thyroid parenchyma could certainly contribute to improving the clinical practice.

The authors of this document appraised critically the topic of how to report the echographic relief of non-nodular thyroid disease as well as non-nodular thyroid parenchyma in the presence of TN. A classification system is here proposed with the intent to (1) cover the current lack of standardized classifications of normal thyroid parenchyma, (2) improve the accuracy of US operators with limited experience in the thyroid field, and (3) facilitate the exchange of information among clinical thyroidologists (endocrinologists as first), thyroid US operators, general practitioners, and other stakeholders.

## Issues and descriptors of thyroid US parenchyma

Ultrasounds can significantly help clinicians to assess the thyroid status of patients with or without thyroid dysfunction, independently of the presence of TN. Indeed, several US parameters can be useful to interpretate various thyroid disorders, and the combination of parameters is much more important than their sole use. Relevant US parameters are listed below, and their significance is defined.

### Echostructure

The thyroid echostructure is expected as by definition homogeneous. Thyroid having homogeneous echostructure means a gland without nodules or parenchyma alterations. Thyroids with tissue alterations should be described with inhomogeneous echostructure.

### Echogenicity

Thyroid echogenicity is usually assessed according to the echogenicity of the near neck muscles. Since normal muscles are always hypoechoic on US, normal thyroid parenchyma is expected to be with higher echogenicity than muscles. In clinical practice, the echogenicity of the submandibular glands is often used as a reference comparable to the echogenicity of normal thyroid [19].

### Vascularization

The thyroid gland is an organ with a rich blood supply and numerous vessels. This can be explored by US-based echo-color doppler analysis. Even if there is no standardized scale to assess the thyroid vascularization, hypervascularization can be observed in some autoimmune as well as inflammatory diseases. As consequence, normal thyroid parenchyma usually presents with low-intensity vascularization. There are recommendations from professional societies regarding the use of doppler- and duplex-US of the thyroid [20].

### Morphological parameters

Ultrasound is recognized as the gold standard to evaluate the thyroid volume. Thyroid size is usually estimated using the ellipsoid volume formula applied to each lobe. This value varies according to individual parameters (i.e., gender and anthropometric characteristics) and can be modified by the presence of TN or autoimmune disorder. The dimensional asymmetry between the two thyroid lobes can be clinically useful to explain eventual symptomatology correlated to tracheal (dyspnea) and/or esophageal (dysphagia) deviation. The shape of the thyroid lobes can also contribute to explaining the visibility/palpability of TN. Lesions adjacent to trachea of esophagus can determine local symptoms (or at least participate to). Nodules localized in the anterior part of the lobe can be visible or at least palpable. As morphological parameters are purely qualitative – being individual and correlated with body measures as body mass index or surface area – and difficultly standardizable, the present document is not focused on these aspects.

## Ultrasound presentation of diffuse thyroid diseases, clinical remarks, and implications

### Autoimmune diseases

The relatively common autoimmune thyroiditis occurs in two forms, a less common hyperplastic form (Hashimoto’s thyroiditis) and a more common chronic-atrophic form (also known as Ord’s thyroiditis), which may be presumed to be expressions of different stages of the disease. Both entities – hyperplastic and atrophic – are often subsumed under the term “Hashimoto’s thyroiditis” in everyday clinical practice. What is striking in these forms of lymphocytic thyroiditis is the equally weaker echogenic echo pattern compared to the normal thyroid, which can regularly (>90%) be detected in later stages of the disease. Typically, there is reduced parenchymal vascularization in chronic lymphocytic thyroiditis, and more frequently increased parenchymal vascularization in the initial phase. The less echogenic thyroid often appears to have been remodeled in a honeycomb manner. Increased thyroid volume can be observed in patients with overt hypothyroidism; this pattern represents the adaptative behavior of the gland to cover the hormones body needs, and it can be reversible after starting replacement therapy with levothyroxine. Circumscribed areas of weaker echogenicity may indicate increased lymphomatous infiltration and make it difficult to differentiate suspicious, ill-defined nodes.

In GD, the thyroid is generally enlarged (i.e., this disease should be formally reported as diffuse hyperfunctioning goiter). The growth tendency causes changes in the depth diameter so that a plump thyroid shape may appear. Characteristic of GD goiter is the change in thyroid size and echo pattern over time. The irregularity of the echo pattern, which can be reversible with treatment, is typical. Anti-thyroid drugs may lead to a decrease in thyroid volume. However, there are no reliable ultrasonographic signs of remission; normalized vascularization is often found at remission, but it cannot be assumed as proof of that and cannot exclude future relapses.

### Inflammatory diseases

Subacute thyroiditis (SAT), also known as “De Quervain”, can affect the entire thyroid or just parts of it. The 3-phase trend of this thyroiditis (disruptive with – generally pauci-symptomatic or asymptomatic – thyrotoxicosis, initial recovery with hypothyroidism, and *restitutio ad integrum*) can be parallelly observed at US; first, parts of the gland are affected, later, all thyroid parenchyma can be injured. Alongside other inflammation features (fever, fatigue, etc.), pain is typical of this thyroiditis and depends on the gland enlargement (particularly the depth diameter) and consequent capsule distention. Pseudonodular areas of strong hypoechogenicity mimicking (high-risk) nodules are frequently observed in the first phase; indeed, differentiation of SAT from malignant nodules can be challenging and, if clinical assessment is not accurately performed, inappropriate patient’s management may occur with unnecessary biopsy and misleading result. Sensitivity to touch and pain caused by the transducer application during sonography are typical, and patients can often localize this precisely. Doppler sonography usually shows reduced vascularization in the inflamed areas. Sonographically detectable enlarged and activated (loss of the more echogenic hilum and the oval shape) cervical lymph nodes are almost always found.

Silent or painless thyroiditis (ST) is also characterized by temporary thyrotoxicosis, occasionally with subsequent transient hypothyroidism, usually followed by complete functional recovery. It accounts for about 0.5–5 percent of cases of thyrotoxicosis. Ultrasonography usually shows a normo-sized to slightly enlarged thyroid, usually slightly to significantly less echogenic; also, it shows atrophic thyroid, which can be considered pre-existing in the context of ATD, even if this is often unknown. Vascularization can be normal or more often significantly increased. Differentiation from disseminated autonomy or antibody-negative GD can occasionally be difficult. However, both are rare in the postpartum period if they were not already known preconceptionally. In addition, autonomies are rare in young people. A scintigraphy can be helpful showing lack of uptake in ST as sign of release thyrotoxicosis without hyperfunction.

Postpartum thyroiditis (PPT) is relatively common, occurring in up to 10% of women 6–12 months after delivery and more often showing a triphasic course with initial thyrotoxicosis, possibly followed by hypothyroidism and usually restitution to euthyroidism. The clinical and sonographic picture is like ST [21, 22].

Riedel’s thyroiditis is a rare form of thyroiditis that consists of a fibrotic process of the thyroid that can cause gland destruction, infiltration of the neck structures, and occasionally obstruction of the airways; recurrent paresis may also occur. No uniform appearance is described in imaging [23, 24]. In most cases the lesions are less echogenic, sometimes very poorly defined or even infiltrating the surrounding tissues, the vascularization is rather reduced, and the elastographic pattern is anelastic. It is not always possible to differentiate between SAT or thyroid carcinoma at US; a core needle biopsy can be useful to avoid a diagnostic surgery.

Checkpoint inhibitors-related thyroiditis (CIRT) are increasingly frequent scenarios in clinical practice. Currently, there are no consistent data on the sonographic thyroid presentation in this context. However, a release thyrotoxicosis is frequent and is only treated symptomatically. A characteristic feature of the CIRT is represented by the unusually rapid transition from thyrotoxicosis to overt hypothyroidism. The sonographic findings in CIRTs can range from largely the normal pattern to typical signs of SAT to changes such as those in ATD. The patient’s clinical history is essential to distinguish CIRT from other inflammation and autoimmune diseases.

Acute infectious thyroiditis and thyroid abscess are rare entities [25, 26]. Due to its capsule, very pronounced blood flow and lymphatic drainage as well as its high iodine content, the thyroid has a natural protection against infections. Sonographically, infectious thyroiditis and abscesses do not present a uniform picture, particularly depending on the stage of the disease, and are therefore also referred to as “chameleons” in diagnostics. In the early stages, inhomogeneous, often poorly defined, sometimes weakly echogenic areas are seen. Solid and isoechoic areas can also occur. Local pressure pain during the examination is typical. Activated lymph nodes are regularly found in the drainage areas. As the inflammation and abscess formation progress, increasingly weakly echogenic areas are found as a sign of melting, possibly with a capsule-like border. The vascularization in the affected areas is reduced and may be increased in the peripheral areas as an inflammatory reaction. Air inclusions as a sign of anaerobic gas-forming bacteria may also be detectable.

## Proposal of classification of patterns

### Thyroid pattern “N” – normal thyroid parenchyma

#### Definition

Thyroid parenchyma with normal echogenicity, homogeneous echostructure, and low/moderate vascularization, independently of the presence of TN.

#### Clinical remarks

Thyroids assessed as “N” are expected normally functioning (i.e., euthyroid patient) and virtually exclude autoimmune disorder (i.e., no need for testing patient for thyroid antibodies). In the presence of TN, patients with non-nodular parenchyma “N” are expected euthyroid with the exception of those having disseminated autonomy that cannot be detected on US.

### Thyroid pattern “A” – autoimmune thyroid disease

#### Definition

Thyroid parenchyma with low or very low echogenicity, inhomogeneous echostructure, presence or not of pseudonodular lesions, and any pattern of vascularization, independently of the presence of TN.

#### Clinical remarks

ATD presents with this typical pattern being GD and HT not fully distinguished ultrasonographically. The pattern of vascularization can be useful to assess the status of autoimmune disease, but not always distinguishes GD from HT. Although GD generally presents at diagnosis with (highly) increased vascularization (“thyroid inferno”), also HT may show increased vascular signal when hypothyroidism is constituted or imminent. Thus, describing hyper-vascularized thyroids as suggestive for GD is discouraged to avoid misleading messages.

### Thyroid pattern “I” – thyroid inflammation

#### Definition

Thyroid parenchyma presenting diffuse hypoechogenicity or shaded area(s) of hypoechogenicity completely devoid of vascularization or echogenic foci, typically in a thyroid with normal echogenicity and normal vascularization with or without TN. Both diffuse and focal hypoechogenicity can be fine or strong. When the inflammation is in an early stage and focal, this pattern can mimic malignant TN.

#### Clinical remarks

Thyroid inflammation include various mechanisms leading to thyroid injury (e.g., infection with direct and indirect involvement of the gland, cytokines-driven inflammation, or drug-induced damage) associated or not with local symptoms (e.g., pressure, pain) that are evocated during the procedure of thyroid US examination. The assessment of the pattern “I” should be completed by these clinical adjunctive features to help clinicians to better address the diagnosis according to the patient specific context.

## Examples of reporting

### Normal thyroid presentation - pattern “N”

**Fig. 1:**
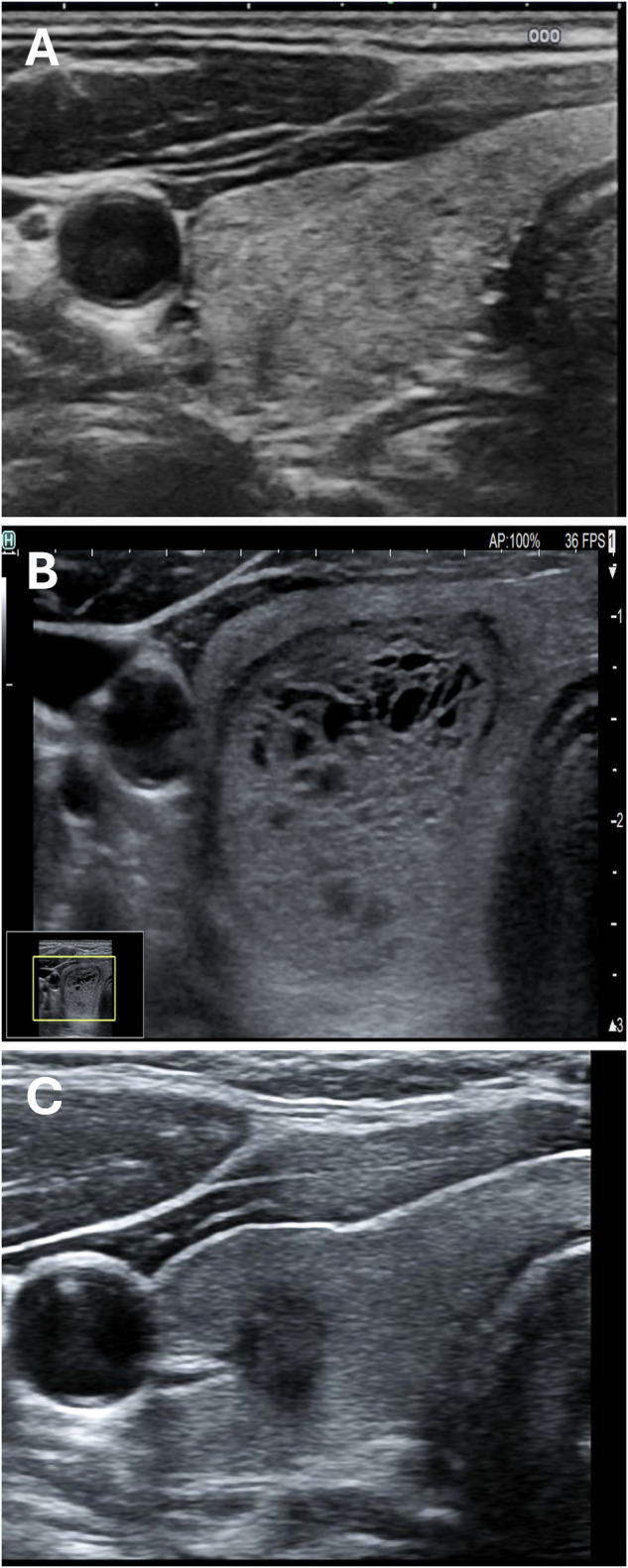
Scenarios of pattern “N”. Normal thyroid parenchyma without nodules **A**. Normal parenchyma with a low-risk nodule **B**. Normal parenchyma with a high-risk nodule **C**
- The thyroid has an estimated size of 17 mL. There are two nodules, one at low risk of 17 mm in the upper right lobe and one at high risk of 7 mm in the lower right lobe. The remaining parenchyma is normal (pattern N). See Fig. 1B, C.

- The thyroid has an estimated size of 12 mL and presents with normal parenchyma (pattern N). See Fig. 1A.

### Thyroid autoimmune disease – pattern “A”

**Fig. 2:**
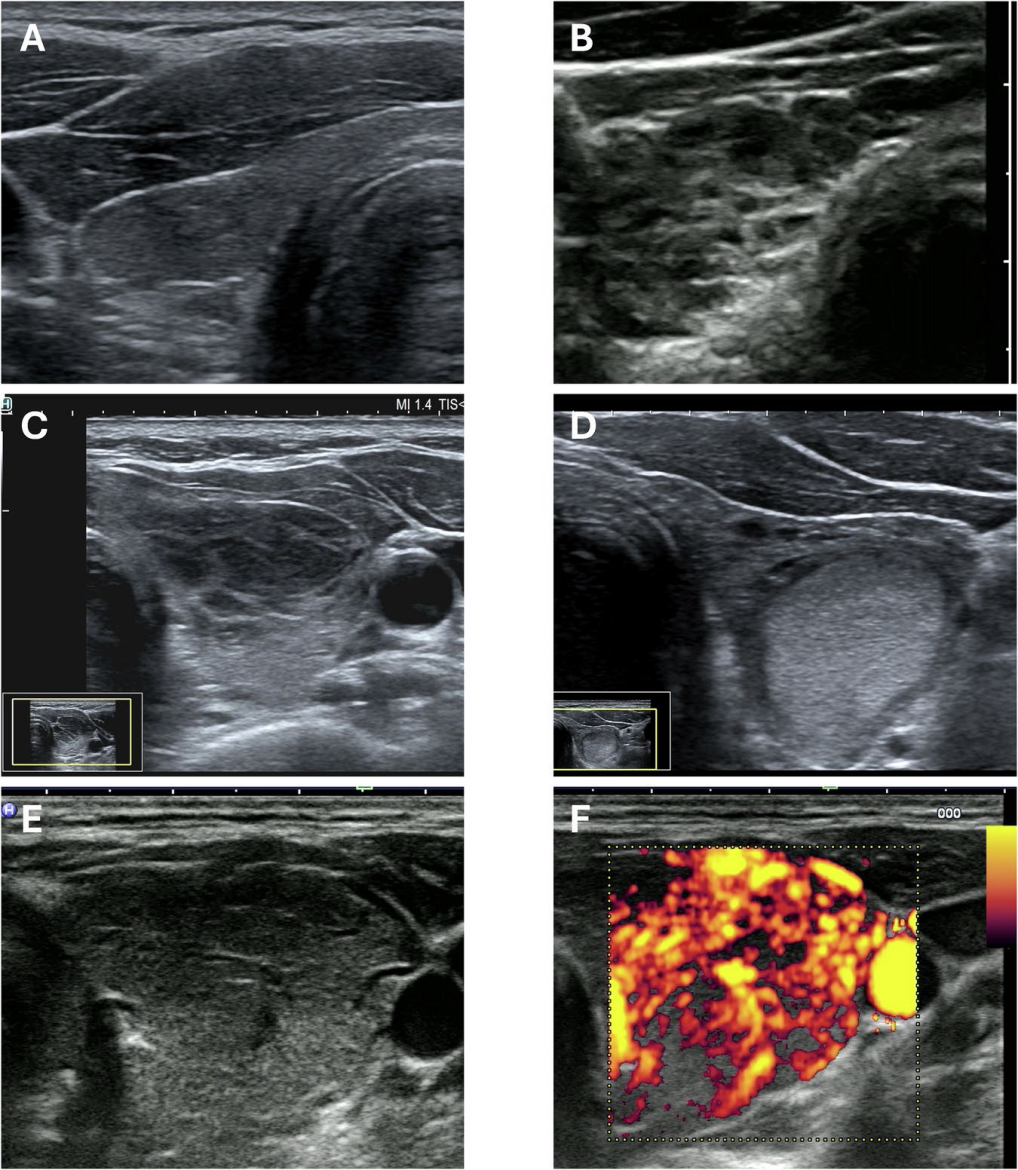
Scenarios of pattern “A”. Homogeneous hypoechoic parenchyma of autoimmune thyroiditis **A**. Inhomogeneous parenchyma of autoimmune thyroiditis with white branches on a black background **B**. Autoimmune thyroiditis with hypoechoic parenchyma in the anterior part of left lobe and normal echogenicity in the remaining part **C**. Normo-echoic nodular lesion (“white knight”) within hypoechoic parenchyma of autoimmune thyroiditis **D**. Hypoechoic parenchyma of Graves’ disease **E** with increased vascularization **F**
- The thyroid has an estimated size of 5 mL with a nodule at low risk (“white knight”) of 10 mm in the left lobe. The remaining parenchyma is abnormal and suggestive for autoimmune disease (pattern A). See Fig. 2D.
- The thyroid has an estimated size of 26 mL with abnormal parenchyma suggestive for autoimmune disease (pattern A) and diffusely increased vascularization. Graves’ or Hashimoto disease is possible, and the patient needs clinical assessment with eventual further specific tests. See Fig. 2E, F.

- The thyroid has an estimated size of 4 mL with abnormal parenchyma suggestive for autoimmune disease (pattern A); see Fig. 2A. In some cases, the parenchyma also appears honeycombed (Fig. 2B). Occasionally, only parts of the thyroid gland are affected by the autoimmune process, in which case normal thyroid tissue can be seen alongside weaker echogenic parenchyma (Fig. 2C).

### Thyroid inflammation – pattern “I”

**Fig. 3:**
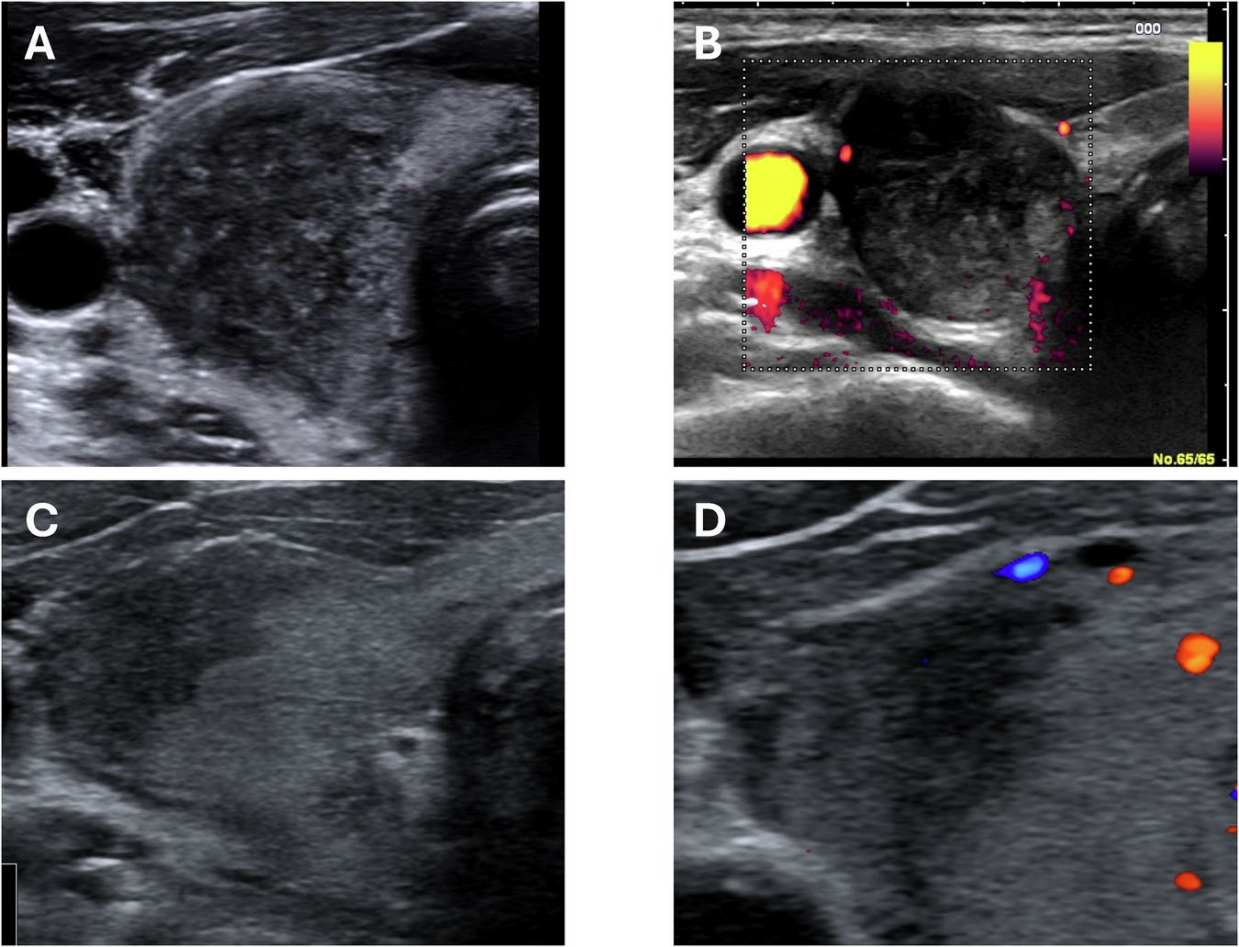
Scenarios of pattern “I”. Shaded hypoechogenicity of silent thyroiditis in the upper part of right thyroid lobe **A**. Thyroid inflammation presenting with marked hypoechogenicity that may mimic a nodule **B**. De Quervain involving lateral part of right thyroid lobe presenting with finely hypoechoic parenchyma **C** and normo-echoic and normally vascularized pattern in the remaining parenchyma **D**

The thyroid has an estimated size of 15 mL. In the upper third of right lobe there is a shaded area of fine hypoechogenicity devoid of vascularization. See Fig. 3A. The inflamed area may also appear more “pseudonodular” (Fig. 3B). The remaining parenchyma has normal echogenicity and normal vascularization. Mild pain is evocated by a slight pressure of the ultrasound probe in correspondence of the right lobe. Pattern I suggestive for subacute thyroiditis to be confirmed in a clinical consultation. See Fig. 3C, D.

## Clinical implications of patterns

Clinical implications of utilizing standardized US-based thyroid patterns could have non negligible implications for clinical practice facilitating the exchange among thyroidologists and stakeholders. Assessing patients with pattern “N” thyroid without TN should imply that thyroid dysfunction is virtually excluded. This can be relevant in patients with incidentally discovered borderline skewed thyroid hormones tests in the absence of specific symptoms. Less-experienced US operators could be facilitated in assessing hypoechoic parenchyma suggestive of ATD with no need for discriminating HT from GD; this can reduce misleading conclusions in favor of HT or GD and improve patient clinical assessment of non-specialized clinicians. Thyroid inflammatory diseases could be defined with better accuracy, improving the understanding by non-specialized clinicians and especially general practitioners who would be aware of the echographic suspicion and prompted to perform patient physical examination. Discriminating ATD from inflammatory diseases might remain challenging when pseudonodular pattern is detected. However, pseudonodular pattern of inflammation disease generally presents with solid defined marked areas mimicking a TN (Fig. 3B) while pseudonodular configuration of ATD usually presents with multiple mildly hypoechoic lesions (Fig. 2B). Hypoechogenicity of these areas is also more pronounced in inflammation disease than in ATD.

Using the proposed US patterns could also improve cost-effectiveness of patient clinical management. Firstly, patients with thyroid assessed as pattern “N” can be judged as euthyroid. Costs for thyroid tests could be saved on, if not retained essential for other reasons. Secondly, US operators evaluating patients with thyroid assessed as pattern “A” can advise for testing thyroid-antibodies, especially in cases when diagnosing autoimmune thyroiditis can change patient management (e.g., female patient is attempting to become pregnant). Thirdly, endocrinologists may manage patients with thyroid inflammation only with routine US saving costs of blood thyroid tests and inflammation marker if not strictly necessary (e.g., cases with discrepancy between clinical signs and US follow-up). In addition, this classification can generally help to reduce unnecessary further diagnostics, such as thyroid scintigraphy.

## Conclusions

According to the aim of this document, the authors hope that the present classification system is implemented in clinical practice and might improve the accuracy of US operators and facilitate the exchange between clinicians and any thyroid US operator. Real-life studies, including various areas with different iodine supply, are mandatory to verify the accuracy of the present classification in prospective trials and eventually improve its ability. Validation trials remain essential before introducing the present classification in clinical practice.

## Data Availability

No datasets were generated or analysed during the current study.
